# Commentary: Clinical evaluation of pediatric olfactory disorders: a review from etiology to management

**DOI:** 10.3389/falgy.2026.1834778

**Published:** 2026-05-20

**Authors:** Gina M. Spencer, Eishaan K. Bhargava

**Affiliations:** 1 Queen's University School of Medicine, Kingston, ON, Canada; 2 Faculty of Health, University of Sheffield, Sheffield, United Kingdom; 3 Department of Otolaryngology, Sheffield Children's Hospital, Sheffield, United Kingdom

**Keywords:** CHARGE syndrome, congenital syndromes, olfactory dysfunction, paediatric rhinology, smell assessment

## Introduction

Trecca and colleagues (1) recently proposed a structured, three-tiered clinical algorithm for the evaluation and management of paediatric olfactory dysfunction (OD) through a mini review. We commend the authors for highlighting an underserved area of paediatric otolaryngology, and for collecting a fragmented evidence base into a digestible framework that is applicable to everyday practice. In brief, their algorithm stratifies evaluation into Tier 1 (detailed medical history and identification of predisposing factors), Tier 2 (psychophysical testing supplemented by subjective report), and Tier 3 (neuroimaging and, where available, electrophysiological assessment).

We write to expand the congenital arm of that framework. Drawing on a recently published systematic review of OD in CHARGE syndrome (CS) as an evidentiary exemplar (2), we extend the discussion to congenital and syndromic paediatric OD more broadly, including Kallmann syndrome, 22q11.2 deletion (3), Bardet–Biedl syndrome (4) and other congenital causes of OD (5), in whom both spontaneous symptom reporting and standard psychophysical testing are unreliable. We identify four concrete extensions to the Trecca et al. framework: (i) proactive olfactory assessment triggered by any congenital or syndromic diagnosis with a known OD association, irrespective of complaint; (ii) a dedicated ‘developmental bypass' branch for children in whom standard testing is not feasible; (iii) a developmental bridge mechanism returning these children to psychophysical assessment as they mature; and (iv) a call for olfactory event-related potential (OERP) technology to transition from a research tool into routine clinical practice for this subgroup.

## Proactive OD case-finding in congenital conditions

First, with respect to prevalence: a 2020 study found that 2/3 of paediatric OD is congenital in nature (6). A 2023 scoping review further identified several common etiologies of pediatric OD, including 22q11.2 deletion syndrome (15.4% of cases of paediatric OD), autism spectrum disorder (12.4%), CS (12.2%), Bardet–Biedl syndrome (6.58%), and Wolfram syndrome (2.98%), many of which may present with associated neurodevelopmental delays (5). Published evidence confirms that OD is highly prevalent across several of these conditions - for example, exceeding 80% in CS when all assessment modalities are considered (2) - making systematic, proactive evaluation essential. We note that, where incidentally diagnosed at high prevalence in a given syndrome, OD may in future warrant consideration within revised diagnostic criteria for those conditions, though this is a consensus matter beyond the scope of the present commentary.

In the flowchart proposed by Trecca et al. (1), congenital conditions including CS are discussed in the manuscript body as examples of “predisposing factors” within the first-level medical history. Whilst clinically accurate, we would caution that this framing risks being passively noted or overlooked entirely by clinicians who do not already associate these conditions with OD. This is of particular concern in children who may never spontaneously report OD due to intellectual disability, limited communicative ability, or absent insight into their own sensory deficits – in whom the symptom cannot be relied upon to trigger assessment. We therefore propose that a confirmed or suspected diagnosis of a congenital or syndromic condition with a recognised OD association - including CS, Kallmann syndrome, 22q11.2 deletion, Bardet–Biedl syndrome - should trigger olfactory evaluation at the first ENT or paediatric encounter, irrespective of whether a smell complaint is volunteered. We note that such children are frequently referred for genetic evaluation, as Trecca et al. describe (1); our recommendation is complementary, ensuring that olfactory assessment occurs at the initial clinical encounter rather than awaiting genetic confirmation.

## Developmental bypass branch: psychophysical testing limitations

We endorse Trecca et al.'s recognition that psychophysical testing may be limited by age, attention span, or comprehension (1). In children with congenital or syndromic conditions and associated neurodevelopmental disability, however, these constraints may be not merely probabilistic but effectively absolute. Children with CS, for instance, frequently present with intellectual disability and developmental delay, prolonged tube feeding, or uncorrected bilateral choanal atresia, each of which can render standard psychophysical assessment [such as UPSIT (7) or Sniffin' Sticks (8)] impractical. Rather than framing these as mere caveats, we propose a formal ‘developmental bypass' criterion: clinicians should move directly to adjunctive assessment - without attempting psychophysical testing - when a child (a) has a non-verbal developmental age below approximately four years, (b) cannot reliably follow a two-step verbal command, (c) has uncorrected bilateral choanal atresia precluding nasal airflow, or (d) is receiving prolonged enteral feeding with no established oral intake. These criteria are offered as practical starting points for clinical discussion, not as validated thresholds.

Even when supplemented by “self” or “parent” evaluation, as discussed by the authors (1), the literature has shown a lack of agreement between parental assessment and psychophysical olfactory testing. For example, Chalouhi et al. in 2005 found that only 6 of 13 children with CS had parental agreement with paediatric adapted Biolfa psychophysical olfactory testing (9). Another study of communication and language development in CS found that objective severity of the condition did not correlate with parental perceptions of their child's impairment (10). Parents may calibrate their assessments against their own expectations or lived experience rather than against normative olfactory function, limiting the reliability of proxy-report as a standalone measure (2). Proxy-report nevertheless remains a useful complement to objective methods and should not be abandoned; its limitations simply reinforce the case for the adjunctive pathway outlined below.

For children routed through the developmental bypass, MRI of the olfactory cleft, anterior skull base, and olfactory bulbs and tracts serves as an important adjunctive investigation, primarily to identify or exclude structural malformations and developmental anomalies relevant to clinical management (1). We agree with Trecca et al. that MRI findings must be integrated with, rather than substituted for, clinical and functional data. As a cautionary note, Weiss et al. demonstrated rare cases of normal olfaction despite apparent olfactory bulb agenesis on MRI (11); although such cases are exceptional and do not diminish MRI utility, they do counsel against inferring functional status from morphology alone.

This has implications beyond CS alone. The diagnostic pathway currently does not include a dedicated branch for children in whom standard psychophysical testing is not feasible due to neurodevelopmental factors—a group that, based on the Payandeh et al. scoping review, may account for at least 40% of congenital paediatric OD cases (5). We propose that a dedicated developmental bypass branch be added to the algorithm for this subgroup. Within this branch, the pathway should direct clinicians toward: (i) a heightened index of suspicion at history and examination; (ii) adjunctive MRI in selected cases to characterise structural anomalies; (iii) proxy-report interpreted in full awareness of its documented limitations; and (iv) OERPs, which offer objective, non-volitional assessment of olfactory pathway function and are particularly well-suited to children who cannot participate in standard testing. Critically, this branch should function as a developmental bridge: as children mature cognitively, or as validated developmentally appropriate psychophysical tools become available, the pathway should direct clinicians back toward psychophysical assessment.

## Discussion

Both papers converge upon several key priorities for the field. Paediatric-specific, developmentally appropriate psychophysical tests validated for children with cognitive and sensory co-impairments are urgently needed; this need extends beyond congenital syndromes, as olfactory abnormalities are increasingly recognised in children with autism spectrum disorder and other neurodevelopmental conditions (12, 13). Longitudinal studies are required to characterise the natural history of OD in syndromic conditions and to evaluate whether early intervention - including safety counselling, targeted sensory approaches exploiting trigeminal co-stimulation (14), or emerging technologies such as olfactory implants (15) - can alter functional and quality-of-life outcomes. A priority deserving particular emphasis is the clinical translation of OERPs, that offer objective, electrophysiological assessment of olfactory pathway function not requiring volitional participation, making them uniquely suited to children with intellectual disability or severe developmental delay - precisely the population in whom standard testing fails. Although OERPs are referenced in both the current commentary and Trecca et al.'s framework, they remain largely confined to specialist research settings. We call on the field to prioritise standardisation of OERP recording protocols for paediatric populations, development of normative paediatric reference ranges, and provision of guidance on clinical indications so that OERPs may become a routine diagnostic option. Investment in portable, accessible OERP technology would be a meaningful step toward equity in diagnosis for the most diagnostically vulnerable children with congenital OD. Incorporating OD into multisensory quality-of-life frameworks and integration into routine assessment alongside vision, hearing, and endocrine screening, would represent a further meaningful advancement in the care of these patients.

In summary, the framework proposed by Trecca and colleagues is valuable and timely. We suggest that its congenital arm could be strengthened by explicitly accommodating children with confirmed or suspected congenital and syndromic OD in whom symptom reporting and standard psychophysical testing are unreliable. Achieving diagnostic equity for this population will require not only algorithmic refinement but also the clinical translation of objective toolslike OERPs, to bring objective olfactory assessment within reach of children who need it most.
